# Survey of Saliva Components and Virus Sensors for Prevention of COVID-19 and Infectious Diseases

**DOI:** 10.3390/bios11010014

**Published:** 2020-12-31

**Authors:** Priya Kishor Dave, Roberto Rojas-Cessa, Ziqian Dong, Vatcharapan Umpaichitra

**Affiliations:** 1 Networking Research Laboratory, Department of Electrical and Computer Engineering, New Jersey Institute of Technology, Newark, NJ 07102, USA; pd386@njit.edu; 2 Department of Electrical and Computer Engineering, New York Institute of Technology, New York, NY 10023, USA; ziqian.dong@nyit.edu; 3 Department of Pediatrics, State University of New York (SUNY) Downstate Health Sciences University, Brooklyn, NY 11203, USA; Vatcharapan.Umpaichitra@downstate.edu

**Keywords:** saliva sensor, human saliva, COVID-19, virus detection, multi-modal saliva detection, droplet detection, humidity sensor, virus sensor, SARS-CoV-2 detection

## Abstract

The United States Centers for Disease Control and Prevention considers saliva contact the lead transmission mean of severe acute respiratory syndrome coronavirus 2 (SARS-CoV-2), which causes the coronavirus disease 2019 (COVID-19). Saliva droplets or aerosols expelled by sneezing, coughing, breathing, and talking may carry this virus. People in close distance may be exposed directly to these droplets or indirectly when touching the droplets that fall on surrounding surfaces and ending up contracting COVID-19 after touching the mucosa tissue of their faces. It is of great interest to quickly and effectively detect the presence of SARS-CoV-2 in an environment, but the existing methods only work in laboratory settings, to the best of our knowledge. However, it may be possible to detect the presence of saliva in the environment and proceed with prevention measures. However, detecting saliva itself has not been documented in the literature. On the other hand, many sensors that detect different organic components in saliva to monitor a person’s health and diagnose different diseases, ranging from diabetes to dental health, have been proposed and they may be used to detect the presence of saliva. This paper surveys sensors that detect organic and inorganic components of human saliva. Humidity sensors are also considered in the detection of saliva because a large portion of saliva is water. Moreover, sensors that detect infectious viruses are also included as they may also be embedded into saliva sensors for a confirmation of the presence of the virus. A classification of sensors by their working principles and the substances they detect is presented, including the sensors’ specifications, sample size, and sensitivity. Indications of which sensors are portable and suitable for field application are presented. This paper also discusses future research and challenges that must be resolved to realize practical saliva sensors. Such sensors may help minimize the spread of not only COVID-19 but also other infectious diseases.

## 1. Introduction

The COVID-19 pandemic has claimed hundreds of thousands of lives, disrupted day-to-day human activity all around the world, and affected local and global economies [1]. While scientists and health-care professionals are still learning about it, this virus has proved to be highly contagious and deadly in some cases [2]. Therefore, there is an effort to develop and test vaccines to reduce the number of infections of this disease. The U.S. Centers for Disease Control and Prevention (CDC) has suggested that COVID-19 mainly spreads from person to person through saliva droplets [3]. These droplets are expelled by coughing, sneezing, talking, or even breathing. They fall on surfaces that people may touch, and eventually bring them in contact with their mucosa tissue at their nose, mouth, or eyes by touching their face. They may eventually contract COVID-19 after the virus enters their system. Moreover, there are also concerns that airborne droplets can be suspended in the air for several minutes [4]. Such airborne droplets may spread very effectively as they can enter the nose when a person breaths air. Currently, SARS-CoV-2 can be detected in a patient after he or she is exposed to the virus and contracts COVID-19.

A sensor that can quickly and accurately detect SARS-CoV-2 in an ad-hoc environment before a person is exposed to it is desirable, but a system that can achieve this has not yet been reported. The complexity of SARS-CoV-2 detection and the lack of knowledge about the virus lead us to wonder how to determine whether an environment presents a high risk for contracting COVID-19 for its occupants. In such case, one can detect the presence of human saliva and take preventative measures. The detection of saliva in an environment may not ensure the presence of the virus but it may indicate the need for taking septic measures that may minimize the possibility of infection. Therefore, this paper focuses on the detection of the presence of saliva. Portable and robust sensors that detect SARS-CoV-2 in the field seem to be complex to realize at this time. On the other hand, there has been interest in detecting different saliva components for health monitoring and disease diagnosis in recent years. Such sensors may identify a saliva droplet by detecting one or a few of its components.

Saliva is a complex mixture of components secreted by three major salivary glands, namely the submandibular, parotid and sublingual glands, and other small parts of the mouth that also produce saliva but in small quantity, such as the gingival fold and oral mucosa [5,6]. The components of human saliva include water, organic proteins, such as amylase, peroxidase, lysozyme, cortisol, and mucin, glucose, cholesterol, urea, and inorganic components or electrolytes [7,8]. The concentration of these components has been used as an indicator or an auxiliary means of diagnosis of various diseases [9,10,11]. Saliva-producing glands have been shown to host SARS-CoV-2 in individuals with COVID-19 while being asymptomatic [12]. Saliva tests for COVID-19 have received recent emergency approval as a non-invasive COVID-19 test by the U.S. Food and Drug Administration (FDA) in May (COVID-19 molecular laboratory developed test, LDT, by Rutgers Clinical Genomics Laboratory) [13] and August (SalivaDirect COVID-19 diagnostic test by Yale School of Public Health) [14], 2020.

This paper presents a survey of sensors that detect or measure the different components of human saliva. These sensors target the detection of organic or inorganic components of saliva. The sensors are classified and presented based on their working principle, construction, and properties, such as sensitivity and the sample size needed for performing the detection. Considering the need to detect specific viruses, including SARS-CoV-2, this paper also presents the existing sensors that detect different viruses that cause upper respiratory disease and spread through saliva, such as influenza, Middle East Respiratory Syndrome (MERS), and SARS-CoV-2. However, these sensors may not be portable enough for their use in the field. This survey also discusses remaining challenges for future research that could realize the design and implementation of saliva sensors.

The organization of this paper is as follows: Section 2 introduces the components of human saliva, their basic functions and concentrations. Section 3 presents the sensors proposed in the surveyed literature that detect both organic and inorganic components of human saliva and categorizes them based on their working principles. Section 4 presents the surveyed humidity sensors. Section 5 presents the surveyed sensors for virus detection. Section 6 discusses whether the presented sensors are potential candidates for the implementation of saliva sensors, challenges, and future work. Section 7 presents our conclusions.

## 2. Components of Saliva

**Figure 1:**
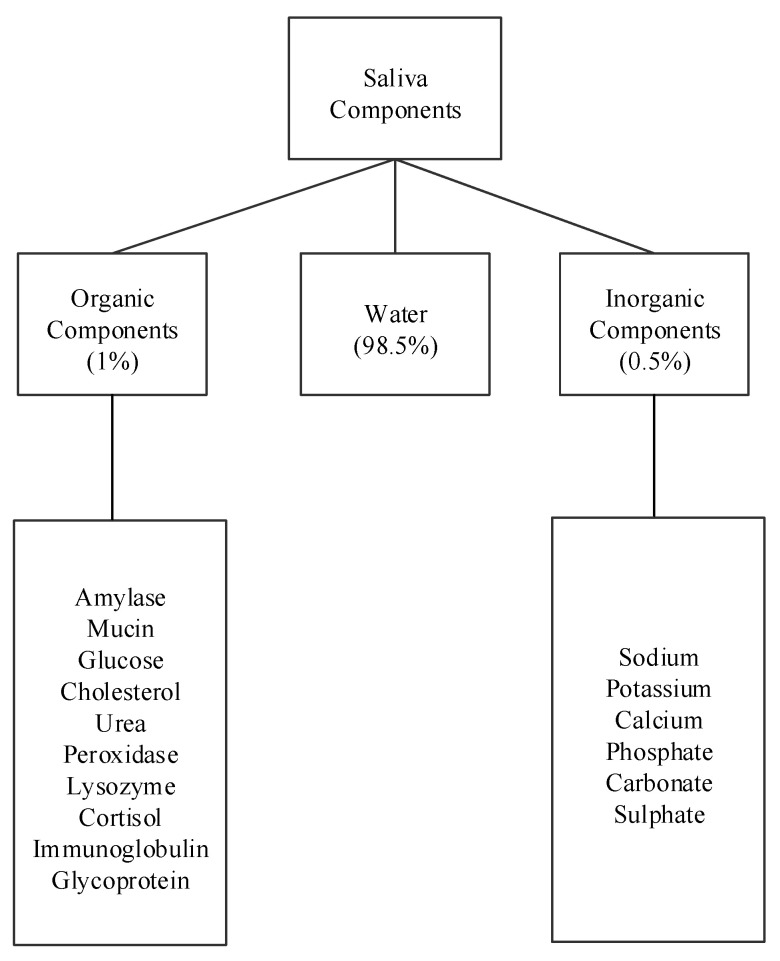
Components of human saliva.

Human saliva comprises water (98.5%), organic (1.0%), and inorganic (0.5%) components as shown in Figure 1. Organic components include proteins, enzymes, mucin and nitrogenous products, such as urea, generated by the human body. Inorganic components are a variety of electrolytes found in saliva and the human body. While the daily average flow of saliva is between 1 to 1.5 L, the concentration of the organic and inorganic components in saliva is small. The small concentrations of these components in saliva impose challenges for their detection in saliva. For example, some components can be detected in saliva, such as amylase, mucin, glucose, cholesterol, urea, peroxidase, lysozyme, and cortisol, while others, such as immunoglobulin and other glycoproteins are found in saliva in such small concentrations that sensors that detect these components focus on serum obtained from blood [15,16]. Inorganic components detectable in saliva are cations, such as sodium, potassium, and calcium, and anions, such as chloride, phosphate, and sulphate.

Table 1 shows the concentrations of saliva components in μg/mL and their percentage compared to other components. Among the listed components, amylase (42%), mucin (17%), and urea (17%) appear in higher concentrations, while cholesterol and cortisol appear in the smallest concentrations. The remaining concentration are other peptides. The remainder of this section describes these saliva components and some of their properties and uses.

### 2.1. Organic Components

#### 2.1.1. Amylase

Amylase is a major digestive enzyme of human saliva. Eighty percent of amylase is synthesized in the parotid glands while 20% in the submandibular glands. It is found in abundance, accounting for as much as 40–50% of the total salivary gland protein [7,17]. Amylase is found with about 60–120 mg/dL in parotid saliva and 25 mg/dL in submandibular saliva.

#### 2.1.2. Cholesterol

Cholesterol (C${}_{27}$H${}_{46}$O) is an essential lipid and the most crucial component for functioning of the human body [7]. The average amount of cholesterol in saliva is 13.56 μg/dL. An elevated level of cholesterol can significantly increase the risk of arterial diseases, coronary heart disease, arteriosclerosis, hypertension, and can be associated with diabetes.

#### 2.1.3. Cortisol

Cortisol (C${}_{21}$H${}_{30}$O${}_{5}$) is a steroid hormone. It is produced as part of the body’s stress response and is secreted by adrenal glands under the control of the hypothalamic-pituitary-adrenal (HPA) system. The quantity of cortisol is an important indicator of psychological stress and for health monitoring [7]. The concentration of cortisol in saliva is from 10 to 20 μg/dL.

#### 2.1.4. Glucose

Glucose (C${}_{6}$H${}_{12}$O${}_{6}$) is usually found in saliva in amounts that range between 0.5 to 1.0 mg/dL. A high concentration of glucose can weaken the immune system and human body can be more prone to diseases [7]. High concentration of glucose also increases the risk of diabetes. Measuring glucose level in saliva has been of interest to monitor and control diabetes as such a test would substitute the more invasive blood test.

#### 2.1.5. Lysozyme

Lysozyme (C${}_{36}$H${}_{61}$N${}_{7}$O${}_{19}$) acts as a booster for the immune system [7,18]. It can attack the polysaccharide wall of bacteria, thus serving as a guard that protects a person from bacterial infection. The quantity of lysozyme found in saliva is from 3.5 to 9.5 μg/mL.

#### 2.1.6. Mucin

Sputum specimens contain saliva, serum transudate, and glycoproteins [7]. Mucus glycoproteins (mucin) are good mediums for bacterial growth and are responsible for the viscous properties of mucus. As a result, characterization and rapid screening of mucus could be used for early diagnosis of chronic obstructive pulmonary disease (COPD). Concentration of Mucin in saliva is about 200 μg/mL.

#### 2.1.7. Peroxidase

Salivary glands secrete a peroxidase enzyme (salivary peroxidase) as well as the thiocyanate ion (SCN${}^{-}$) [7]. The enzyme catalyzes the oxidation of SCN${}^{-}$ by hydrogen peroxide (H${}_{2}$O${}_{2}$). Oxidized forms of SCN${}^{-}$ temporarily inhibit the growth, respiration, and metabolism of most species of oral bacteria [19]. Peroxidase contributes around 15 to 20% of the total amount of saliva.

#### 2.1.8. Urea

The concentration of salivary urea may reflect renal damage. It is used to monitor kidney function in CKD patients, and helps in the diagnosis of middle- and late-stage CKD [7]. Urea (CH${}_{4}$N${}_{2}$O) is found in saliva from 12 to 20 mg/dL.

### 2.2. Inorganic Components

Inorganic components of saliva include a variety of electrolytes, such as sodium, potassium, calcium, chloride, phosphate, sulphate, etc. Electrolytes play a critical role in regulating fluid levels in the body and the blood plasma, maintaining the pH levels of the blood, enabling muscle contractions, transmitting nerve signals from muscles, heart, nerve cells, etc. Electrolytes need to be balanced for the body to function properly. The concentration of electrolytes in saliva is 129.2 mmol/L. Determination of electrolyte composition in saliva is commonly used in the monitoring of dental caries [7].

### 2.3. Water

Water constitutes about 98.5% of human saliva. An indirect method to detect the presence of human saliva in an indoor environment is by measuring the amount of water on a surface or humidity in the air. In fact, water carries the organic and inorganic components of human saliva so the presence of water may indicate the presence of saliva.

## 3. Sensors for Organic and Inorganic Components of Human Saliva

Many of the organic components of human saliva, such as immunoglobulin, glycoprotein, glucose, cholesterol, etc., are also found in blood and have been used as indicators to monitor a person’s health. Therefore, there is an interest in detecting and measuring their concentration in saliva.

**Figure 2:**
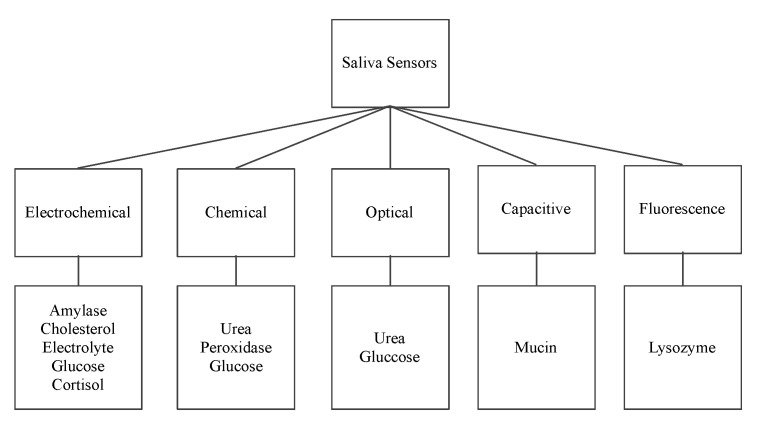
Saliva sensors and their working principle.

The following sensors are designed to detect the organic salivary components and measure their concentration. The paper presents a classification of sensors according to their working principle, target component, and technology used. Figure 2 shows the surveyed salivary component sensors and their different detection working principles: electrochemical, optical, chemical, capacitive, and fluorescence.

### 3.1. Electrochemical Sensors

Electrochemical sensors involve a transfer of charges due to spontaneous reaction that takes place in the electrolytic solution when a suitable voltage is applied between the Working Electrode (WE) and the Reference Electrode (RE) [20,21,22,23,24,25]. This transfer of charges results in output current between the WE and the Counter Electrode (CE) corresponding to the change in the concentration of a specific component.

#### 3.1.1. Enzyme Modified Alpha Amylase Sensor

Zheng et al. [20] proposed a biosensor that detects alpha-amylase using electrochemical working principle. During the detection of amylase, a KCl-saturated calomel electrode (SCE) is used as the reference, a platinum wire as the counter electrode, and an enzyme modified screen-printed electrode as the working electrode. The starch solution acts as the substrate. The measured output is the current generated by applying different voltages. This sensor uses encapsulation of both glucose oxidase (GOD) and α-glucosidase in a composite film of sol–gel and Nafion. Here, Nafion immobilizes 1-dimethyl-3-2-amino-1-hydroxyethyl ferrocene (DMAHFc), as an electron transferring mediator, on the surface of the screen-printed electrode. DMAHFc, sol–gel, and Nafion enhance electron transfer, and improve the sensitivity and long-term stability of the biosensor. This electrochemical sensor has a detection limit of 10 to 656.6 mg/L. The detection limit is the minimum and maximum amounts of the component under test that can be measured.

#### 3.1.2. Platinum Nano-Cluster Combination as a Cholesterol Sensor

**Figure 3:**
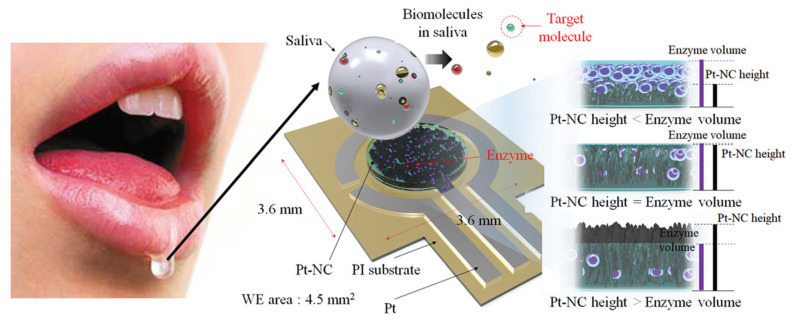
Biosensor with a Pt nano-cluster and strategy on changing the concentration of enzyme over the surface area of the substrate may improve sensor’s performance by optimizing the effective surface area and enzyme volume [21].

Eom et al. [21] proposed a platinum nano-cluster combination (Pt-NC) sensor that detects cholesterol. For the detection of cholesterol, a three-electrode cell configuration is used where Pt-NC, enzyme (mixture of cholesterol esterase, cholesterol oxidase, and peroxidase), and Nafion (Pt-NC/enzyme/Nafion). Nafion acts as the working electrode, Pt as the counter electrode, and Ag/AgCl as the reference electrode. This is an enzyme-based biosensor that detects low concentrations of cholesterol in saliva. The biosensor exhibits a linear response in the range of 2 to 486 μM and a detection limit of about 2 μM. The reported sensitivity of the sensor is 132 μA/mMcm${}^{2}$. Figure 3 shows the Pt-NC sensor for cholesterol detection.

#### 3.1.3. Immunosensor: Interdigitated Microelectrodes-Based Cortisol Sensor

Pasha et al. [22] proposed a simple, low-cost, label-free, electrochemical immunosensing platform for a highly sensitive and selective detection of cortisol in saliva. Electrochemical immunosensing platform has been utilized for a low-cost and label-free detection of cortisol via covalent immobilization of anti-cortisol antibodies (Anti-Cab) on self-assembled monolayer (SAM) of dithiobis succinimidylpropionte (DTSP) with modified microfabricated interdigitated microelectrodes (IDEs). The sensor is binded using ethyleneamine (EA). The non-binding sites of the immunosensor’s surface are blocked using EA. The concentration of cortisol produces an electrochemical response using cyclic voltametry. The sensor exhibits a detection that ranges from 10 pg/mL to 100 ng/mL, a detection limit of 10 pg/mL, and a sensitivity of 6 μA/(pg/mL), with a regression coefficient of 0.99.

#### 3.1.4. Antibody Modified Gold Microarray Electrode Cortisol Sensor

**Figure 4:**
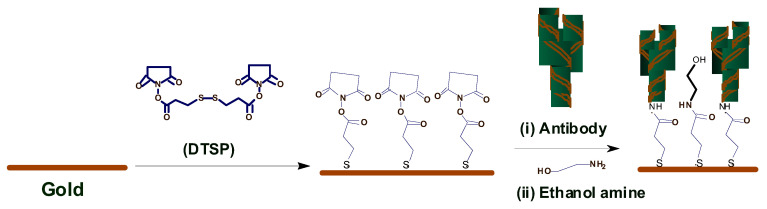
Ethyleneamine (EA)/C-Mab/dithiobis succinimidylpropionte (DTSP)/Au bio-electrode [23].

Arya et al. [23] proposed a microarray electrode sensor that detects cortisol hormone in saliva. This sensor is similar to the one in Section 3.1.3 with functionalized gold (Au) microelectrode array. This sensor uses an electrochemical impedance spectroscopy (EIS) technique to make cortisol measurements. It monitors the change on resistance by the different currents. The EA/C-Mab/DTSP/Au-based biosensor can accurately detect cortisol in the range of 1 pM to 1 μM with a sensitivity of 1 pM. Figure 4 shows the operation of the EA/C-Mab/DTSP/Au bio-electrode.

#### 3.1.5. Single-Walled Carbon Nanotube Sensor

**Figure 5:**
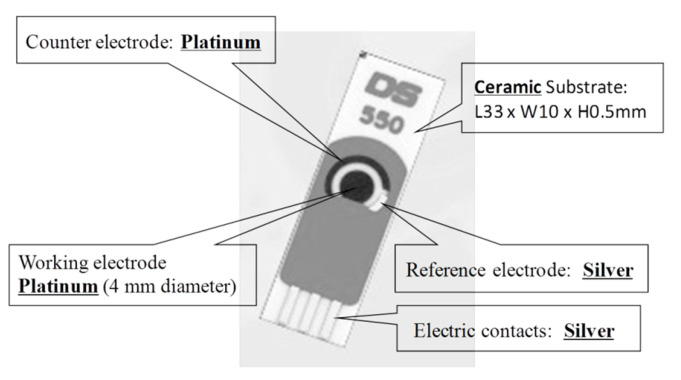
Screen-printed three-electrode glucose sensor [24].

Du et al. [24] proposed a single-walled carbon nanotube sensor to detect salivary glucose. For the detection of glucose three metallic electrodes are used: a Pt WE, a silver (Ag) RE, and a Pt CE. These salivary glucose sensors are built using layer-by-layer self-assembled single-walled carbon nanotubes, chitosan, gold nanoparticles, and glucose oxidase onto a screen-printed platinum electrode. An electrochemical sensor is utilized for a quantitative detection of glucose in both buffer solution and saliva samples. The buffer solution is used as a reference. The WE and CE are used to detect changes of glucose concentrations. Cyclic voltammetry (CV) electro-analytical tests can be conducted with these sensors. In such tests, a suitable voltage is applied between the WE and RE. The current between the WE and CE corresponds to the concentration of glucose. Figure 5 shows this electrochemical glucose sensor and its parts.

#### 3.1.6. Mouthguard Cavitas Sensor

**Figure 6:**
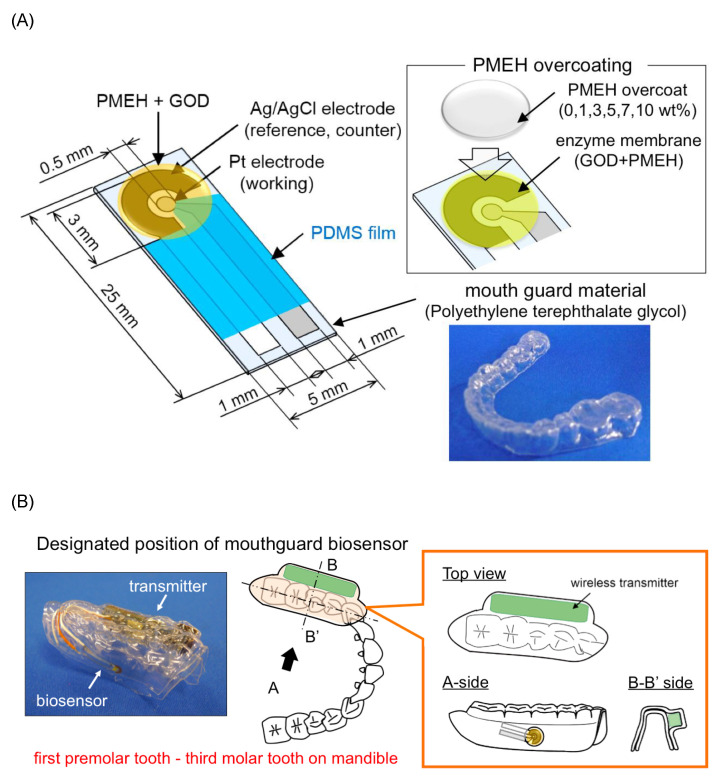
(**A**) Glucose biosensor on the polyethylene terephthalate glycol mouthguard support. (**B**) Mouthguard biosensor to custom-fit the patient’s dentition [25].

Arakawa et al. [25] proposed a mouthguard cavitas sensor to detect salivary glucose. This glucose sensor consists of a platinum and silver/silver chloride electrode (Ag/AgCl), with glucose oxidase (GOD) immobilized by poly2-methacryloyloxyethyl phosphorylcholine-co-2-ethylhexylmethacrylate (PMEH) on a custom-fitted monolithic mouth-guard. It has a wireless transmitter for telemetric measurement of saliva glucose. A 0.5 mm-thick layer of polyethylene terephthalate glycol (PETG) is selected as part of the structure to demonstrate a strong adhesion of Pt and Ag to PETG. This sensor has GOD and a PMEH overcoat applied to the sensing region. PMEH over-coating increases the sensitivity of the sensor. The biosensor is capable of real-time continuous wireless measurement of glucose in artificial saliva from 5–1000 μmol/L with a phantom jaw. Figure 6 shows the mouthguard sensor and its operation.

### 3.2. Chemical Colorimetric Sensors

Colorimetric detection involves the use of substances that react chemically to detect the presence of a specific component [26,27,28]. The following sensors use colorimetric detection.

#### 3.2.1. Colorimetric Urea Sensor

Evans et al. [26] proposed a colorimetric sensor to detect urea in saliva. The change of color on the testpad indicates the presence of Urea. The Salivary Urea Nitrogen (SUN) dipstick method determines the amount of urea in saliva. This method can be applied to unstimulated saliva, from which 50 μL are used to moisten the test pad of a colorimetric SUN dipstick. Unstimulated saliva is obtained from a subject fasting for at least 15 min prior to the sample collection. The change in color of the test pad is assessed after one minute of being moistened. The color of the test pad is compared to the color of the six reference pads corresponding to increasing SUN concentrations: 5 to 14 mg/dL (pad 1), 15 to 24 mg/dL (pad 2), 25 to 34 mg/dL (pad 3), 35 to 54 mg/dL (pad 4), 55 to 74 mg/dL (pad 5), and ≥75 mg/dL (pad 6). This sensor has a sensitivity of 12.82% and a specificity of 97.33% in the detection of acute kidney disease. Another optical portable sensor of urea has also been reported [29].

#### 3.2.2. Colorimetric Glucose Sensor

Agrawal et al. [27] proposed a colorimetric sensor that detects glucose. For glucose detection, glucose oxidase acts as a catalyst and reacts with saliva to generate a red color complex. The quantitative estimation of fasting plasma glucose (FPG) and fasting saliva glucose (FSG) is performed by a glucose oxidase method using enzymatic kit Glucose Oxidase-Peroxidase (GOD-POD). GOD catalyses the oxidation of glucose to gluconic acid. The formed hydrogen peroxide (H${}_{2}$O${}_{2}$) is detected by a chromogenic oxygen acceptor phenol, 4-AP, 4-aminophenazone in the presence of peroxidase. The red-colored complex quinoneimine is measured colorimetrically and the intensity of the color formed is directly proportional to the concentration of glucose in the sample. The mean FSG of the subjects in a non-diabetic group was reported as 6.08 ± 1.16 mg/dL and the mean FSG of subjects in a diabetic group was 10.93 ± 1.93 mg/dL.

There are portable [30,31,32,33] and non portable [29,34,35,36,37,38] sensors of salivary glucose reported in the literature. These sensors are based on chemical, electrochemical and optical working principles.

#### 3.2.3. Chemical Peroxidase Sensor

The salivary peroxidase (SP) system is one of the non-immunoglobulin defense systems in saliva. Vučićević-Boras et al. [28] proposed a sensor that detects peroxidase using a chemical working principle. In peroxidase detection, 3-ethylbenzothiazoline-6-sulfonic acid (ABTS) reacts with saliva to generate a red color complex indicating the presence of peroxidase in saliva. The reagents used for salivary analysis are 20 mM 2.2 azino-di-3-etil-benzotiazolin-6-sulphonic acid diammonium acid (ABTS) in 67 mM phosphate buffer with pH of 6.0 and 10 mM hydrogen peroxide and peroxidase in quantity of 250 J/kg. The assay is based on 0.5 mL of saliva sample, which is diluted with 1.5 mL of phosphate buffer with a pH of 6.0. The reactive mixture consists of 2 mL of diluted saliva, 0.2 mL of ABTS solution, and 0.2 mL of hydrogen peroxide, which are all mixed in a reactive cuvette and analyzed by a spectrometer at 405 nm and at 25 ${}^{\circ }$C. The absorbance becomes red between the first and fifth minutes when using the reagent as a blind trial to indicate the presence of peroxidase.

### 3.3. Optical

Optical sensors either uses colorimetric optical absorption or measure a change in wavelength when there is light absorption by the component [39,40].

#### 3.3.1. Optical Urea Sensor

Soni et al. [39] proposed an optical sensor to detect urea in saliva. This component is detected using colorimetric optical absorption where a strip changes color upon reacting with urea in saliva. The color change is determined using a smartphone application. The sensor is used to detect on sight of kidney disease. The used amount of saliva is 10 μL. The sensor is fabricated using a simple methodology; by immobilizing enzyme urease along with a pH indicator on a filter paper-based strip. The strip changes color upon the reaction of urea in saliva. This color change is determined using a smartphone application; based on Red, Green, and Blue (RGB) profiling. Calibration of the sensor is carried out using saliva spiked with synthetic urea samples with various concentrations. The reported sensitivity of the sensor is 10.9 mg/dL.

#### 3.3.2. Optical Amylase Sensor

**Figure 7:**
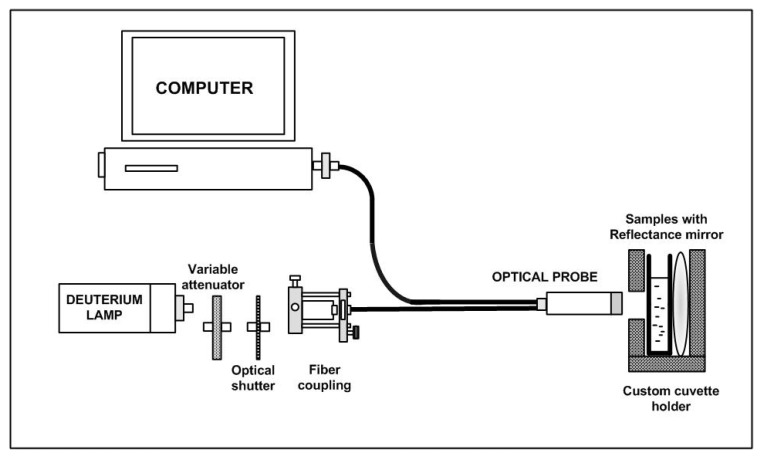
Experimental layout for characterization of saliva samples in the detection of amylase [40].

Yazid et al. [40] proposed an optical sensor to detect dental caries by detecting the amount of amylase in saliva. Amylase can be detected on the basis of optical absorption using an ultraviolet-visible (UV-VIS) spectrometer. The alpha-amylase is absorbed by light at a specific wavelength. When the light at those wavelengths passes through the saliva specimen, some of the light is absorbed by the solute and some other passes through the sample. This difference in the original and transmitted light is called absorbance. The spectrometer detects the absorbance at around 280 nm. The quantity of saliva used for the detection is 2 mL. Six core fibers deliver the UV light to the saliva sample and one fiber in the middle collects the reflected light back to the other end. The sample is placed in a special 350 μL quartz cuvette, which allows the transmission of UV light. The reflected UV light is measured by the high-resolution UV–VIS spectrometer, which is capable of detecting a wavelength range from 200 nm to 1100 nm at 0.01 nm resolution. It is observed that the absorbance of the saliva samples of a healthy group are the lowest as compared to that of a low caries group and high caries group, because of the amylase concentration. Figure 7 shows the optical detection of amylase.

### 3.4. Capacitive

Capacitive sensors are non-contact devices that can detect the presence or absence of virtually any object regardless of material. They utilize the electrical property of capacitance and the change of capacitance based on a change in the electrical field around the active face of the sensor [41,42].

#### Complementary Metal-Oxide Semiconductor Mucin Biosensors

Guha et al. [41] and Soltani et al. [42] proposed two complementary metal-oxide semiconductor (CMOS) biosensors that detect mucin using a capacitive working principle. The operation of the sensors are based on the detection of change in the dielectric constant of the sputum-mucin due to varying viscosity. A planar interdigitated capacitor (IDC) is embedded in a CMOS oscillator, which works as the sensor. The two models considered for estimation and understanding of variation of viscosity are a) glycerol water mixture and b) glycerol ethanol mixture. The reported sensors use CMOS and bi-polar CMOS (BiCMOS) integrated technology. The sensing principle of these two sensors is based on the detection of a dielectric change of the capacitor embedded in a resonant CMOS oscillator. The operating frequency of the sensors is 12 GHz. The sensors can distinguish between a highly viscous clot of sputum-mucin and diluted sputum-mucin.

### 3.5. Fluorescence

Fluorescence detection often includes a source of excitation light (e.g., LED photo-diodes), a fluorophore particle, wavelength filters to isolate photon emissions from excitable molecules, and a detector that records changes in fluorescence intensity and generates a measurable value [43,44].

#### Fluorescence Lysozyme Biosensor

Liu et al. [43] proposed a fluorescence-based sensor to detect the presence of lysozyme in saliva. The anti-lysozyme DNA aptamer labeled with fluorescein (FAM) is used as a probe to recognize and transduce a fluorescence signal. The mixture gets adsorbed on the surface of graphene oxide (GO) via strong binding and hydrophobic interactions between the Single-Stranded DNA (ssDNA) and GO. In this case, the FAM and the acceptor (i.e., GO) come in close proximity. As a result, the intensity of fluorescence decreases due to energy transfer from FAM to GO. When lysozyme comes in contact with the FAM aptamer, it gets combined with FAM aptamer and the resulting FAM-lysozyme structure gets separated from GO. Due to this separation, the intensity of fluorescence is increased and the maximum intensity of the signal is found at a wavelength of 480 nm, indicating the presence of lysozyme. This fluorometric lysozyme sensor has a detection sensitivity of 2 μg/mL with a detection limit of 21.8 pM. The specificity of this sensor is 85%, defined in terms of flurorescence recovery and under the presence of six other proteins as 500 pM along with 250 pM of lysozyme. This sensor does not require cleaning or replacement and it can be used for long periods of time. Khan et al. [44] recently proposed another portable lysozyme sensor. There are other proposed lysozyme sensors that are; however, not portable [45,46].

### 3.6. Sensors for Inorganic Components of Human Saliva

Rumenjak et al. [47] proposed a potentiometric sensor to detect the electrolyte concentration of a substance in saliva. This method determines concurrent concentrations of potassium, calcium, and chloride using ion-selective electrodes. The instrument consists of three ion-selective electrodes and a reference electrode, two peristaltic pumps for collecting samples and washing the electrodes after use, and a computer. Prior to the measurement, the instrument is calibrated using undiluted standard samples containing potassium, calcium, and chloride with different concentrations. The saliva sample is diluted by a magnesium acetate solution at 1:10. The development of this sensor is motivated for the detection of dental problems by determining the calcium concentrations. Urbanowicz et al. [48] also proposed a portable electrolyte sensor to determine the ionic profiles in human saliva.

There are other portable sensors of urea [49], glucose [30,31,32,33], lysozyme [44], and inorganic components [48] reported in the literature that may present some similarities to the ones described above.

## 4. Humidity Sensors

**Figure 8:**
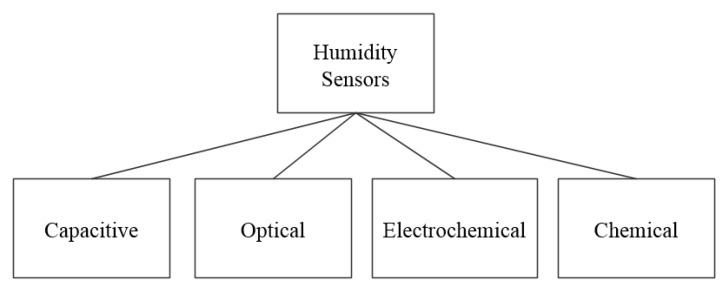
Working principles of surveyed humidity sensors.

As water constitutes the largest portion of human saliva (98.5%), humidity sensors can be used to detect the presence of saliva on a surface. Figure 8 shows the working principles of the humidity sensors, which include capacitive, optical, electrochemical, and chemical. The remainder of this section describes sensors in these different categories.

### 4.1. Capacitive

#### 4.1.1. Capacitive-Based Humidity Sensor

**Figure 9:**
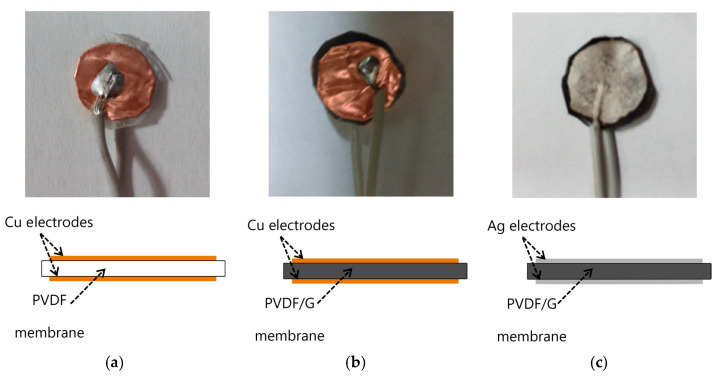
The morphology of a PVDF/G membrane was tested. In this case a layer of silver paint was used as electrode. The samples with electrodes are (**a**) PVDF-Cu; (**b**) PVDF/G-Cu, and (**c**) PVDF/G-Ag samples [50].

Hydrophobicity is one of the important factors in capacitive humidity sensors. The inclusion of graphene (G) in polyvinylidene fluoride (PVDF) improves hydrophobicity. Hernandez et al. [50] proposed the fabrication of electrospun membranes of PVDF blended with G to improve the hydrophobicity property. It allows the use of a PVDF/G membrane as a capacitive humidity sensor. Electrospun membranes offer high porosity, uniform pore size with a narrow pore-size distribution, and a large surface area-to-pore volume ratio. The PVDF/G membrane has a faster response than commercial DHT11 sensors. Figure 9 shows three samples of capacitive humidity sensors, each made with different membrane materials: (a) PVDF-Cu; (b) PVDF/G-Cu, and (c) PVDF/G-Ag electrodes.

#### 4.1.2. Alumina-Based Cross-Capacitive Humidity Sensor

Zargar et al. [51] proposed a cross-capacitive humidity sensor. To fabricate this sensor, a thin film of porous alumina (γ-Al${}_{2}$O${}_{3}$) is deposited on the inner wall of the quartz tube for absorbing humidity. The four symmetrical silver electrodes of the cross-capacitor are formed around the quartz tube by a screen-printing technique. The sensor has been tested for different levels of humidity in the range of 0–90% relative humidity (RH). The capacitive response of the fabricated sensor is very accurate (±2% RH), highly repeatable (±0.01%), with very low hysteresis (±0.3%), and a small drift. Thus, the sensor can be used for making a hygrometer for commercial applications. The main advantage of the cross-capacitor is the single-dimensional accuracy. Depositing the thin sensing film on the inner surface of the quartz tube improves the sensitivity of the sensor.

#### 4.1.3. Multi-Walled Carbon Nanotube Capacitive Humidity Sensor

**Figure 10:**
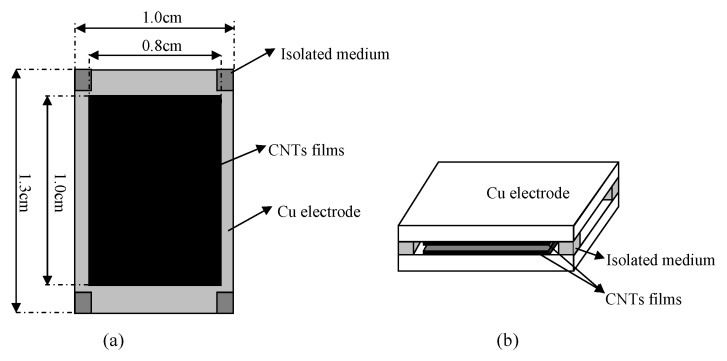
Detailed structure feature (**a**) and schematic diagram (**b**) of a capacitive humidity sensor [52].

Chen et al. [52] proposed a multi-walled carbon nanotube capacitive humidity sensor. Figure 10 shows the structure and schematic of this sensor. This sensor consists of two plate electrodes coated with Multi-walled carbon nanotube (MWCNT) films and four pieces of isolating medium at the four corners of the sensor. According to capillary condensation, the capacitance signal of the sensor is sensitive to RH, which could be transformed to voltage signal by a capacitance to voltage converter circuit. The sensor is tested using different saturated saline solutions at ambient temperature (25 ${}^{\circ }$C), which yields approximately 11 to 97% RH.

### 4.2. Optical

#### 4.2.1. Tungsten Disulfide Sensor

**Figure 11:**
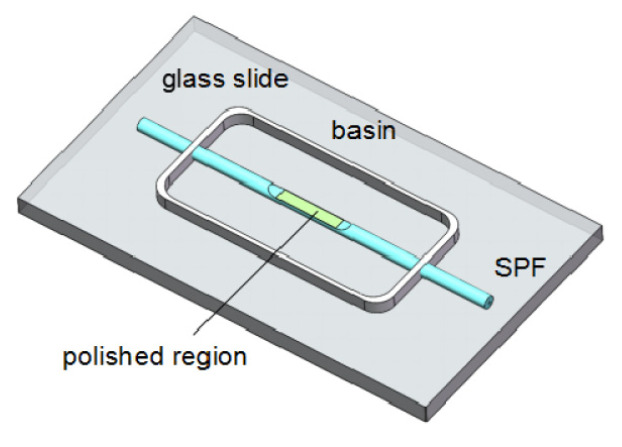
Diagram of a fiber-optic humidity sensor [53].

Luo et al. [53] proposed a fiber-optic humidity sensor that uses tungsten disulfide (WS2), which is combined with side-polished fibers (SPF). Figure 11 shows the optical humidity sensor. Using WS2 alcohol suspension, a film is coated on the polished surface of an SPF through deposition. Its simple and inexpensive fabrication, compatibility with a fiber-optic system, and its flat polished surface make SPF an ideal candidate for combining it with WS2. A 1550-nm laser is coupled to three different fiber samples: unpolished single-mode fiber (SMF), SPF, and WS2-coated SPF (WS2CSPF). SMF is used to monitor the power fluctuation of the laser, and SPF is arranged for control measurements. This sensor works in a RH range of 35–85%.

#### 4.2.2. Tin Oxide-Doped Optical Sensor

**Figure 12:**
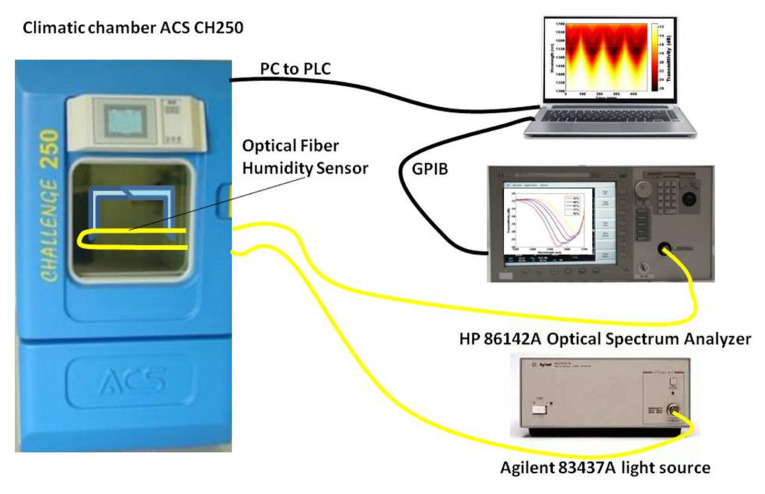
Experimental setup for the tin oxide-doped optical sensor [54].

Ascorbe et al. [54] proposed a high sensitivity optical fiber humidity sensor (OFHS), which is fabricated using a cladding-etched single mode optical fiber (CE-SMF) coated with a thin film of tin oxide (SnO${}_{2}$). Figure 12 shows the optical humidity sensor. The etching is made using hydrofluoric acid and the coating has been fabricated by sputtering. Tin oxide is used to build the nano-coating and works as sensitive material. The device is tested using a climatic chamber in order to obtain the response of the OFHS to relative humidity. Changes in wavelength greater than 130 nm have been obtained for relative humidity varying from 20 to 90%.

#### 4.2.3. Agarose-Doped Polymethyl Methacrylate (PMMA) Sensor

Irawati et al. [55] proposed an optical humidity sensor based on the polymethyl methacrylate (PMMA) microfiber-doped agarose. The doping of agarose gel is enabled by the use of PMMA. Agarose gel is a highly attractive material as it is stable, non-water soluble, and is commonly used in other sensing applications. The humidity sensor relies on a humidity-induced refractive index change in the agarose gel. As a result, the transmitted optical power through the PMMA doped agarose gel microfiber varies as a function of the RH. This sensor operates optimally within the RH range of 50 to 80%, with a sensitivity of 0.1 μW/%.

### 4.3. Electrochemical

#### Single-Walled Carbon Nanotube-Based Humidity Sensor

**Figure 13:**
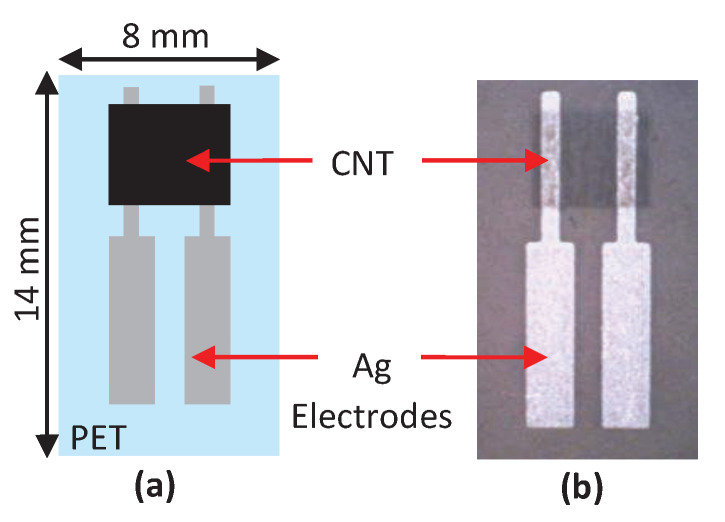
(**a**) Detailed structure feature of humidity sensor and (**b**) photography of fully printed humidity sensor [56].

Turkani et al. [56] proposed a multilayer electrochemical humidity sensor. Figure 13 shows the proposed humidity sensor. This sensor was fabricated using additive print manufacturing processes on a flexible polyethylene terephthalate (PET) substrate. Two silver (Ag) electrodes are deposited using screen printing process. Gravure printing process is used to deposit a carbon nanotube (CNT) sensing layer between the Ag electrodes. The performance of the fabricated sensor is evaluated by measuring its response towards varying RH ranging from 20 to 80%, with increments of 10%. The resistive response of the humidity sensor exhibits a linear response over the entire operational RH range resulting in an overall resistance change of 6%. Here, the sensitivity of the sensor is measured as $\left(1/R_{0}\right)\left(\Delta R/\Delta \%RH\right)100\%$, where $R_{0}$ is the base resistance at 20% RH and ΔR/Δ%RH is the slope of the linear response. A sensitivity of 0.1%/%RH and a correlation coefficient of 0.9484 are reported.

### 4.4. Chemical

#### Electroceramic-Based Sensor

**Figure 14:**
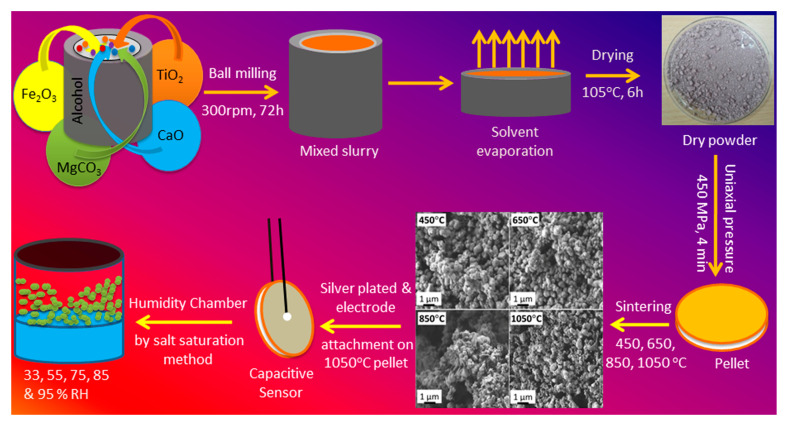
Flow chart for sensor fabrication with the morphology at different sintering temperatures [57].

Tripathy et al. [57] proposed a novel submicroporous Ca, Mg, Fe, Ti-Oxides (CMFTO) electroceramic-based capacitive humidity sensor. This sensor is fabricated from oxide nanomaterials using a solid-state step-sintering process, as shown in Figure 14. The sintering technique defines the desired morphology, lower density, and high porosity of nanomaterials. This Pb-free CMFTO electroceramic and capacitive humidity sensor uses physisorption to improve the sensing properties. The sensor can operate at two humidity levels; one at the lower range of 33 to 75% and the other at the higher range of 85 to 95%. The RH changes from 33 to 95% at a testing frequency of 102 Hz. It achieves high sensitivity (3000%), rapid response (14.5 s) and recovery (34.27 s).This sensor also achieves high linearity and stability of the CMFTO electroceramic. Those features indicate that this sensor can be used as a potential humidity sensing material for advanced applications.

## 5. Sensors for Virus Detection

**Figure 15:**
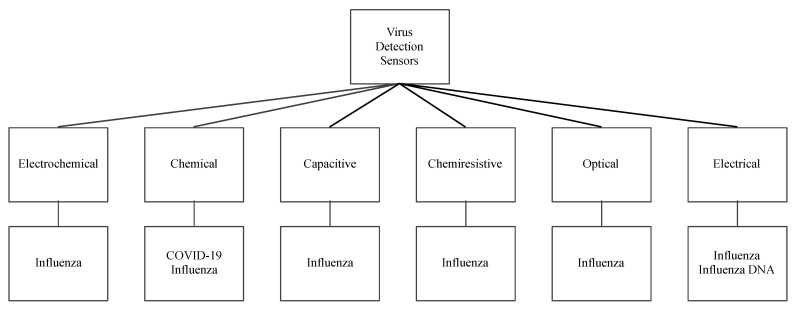
Virus sensors and their working principles.

While saliva sensors present an opportunity to detect the possibility of unveiling an infectious agent, there has been interest in directly detecting specific viruses. Therefore, we also surveyed some of the sensors that detect influenza and other coronaviruses that spread through saliva droplets, such as SARS-CoV-2. Figure 15 shows a classification of these virus sensors according to their working principle, detection elements, and sensitivity. They are also classified into chemical, electrochemical, capacitive, chemiresistive, optical, and electrical sensors.

### 5.1. Electrochemical Virus Sensors

#### 5.1.1. Paper-Based H1N1 Influenza Sensor

**Figure 16:**
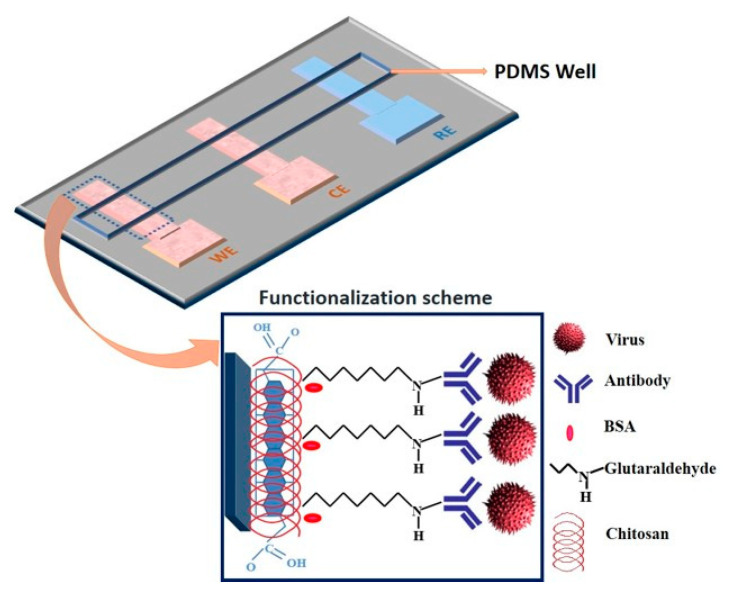
Paper-based immunosensor with a polydimethylsiloxane well containing the electrolyte [58].

Devarakonda et al. [58] proposed an electrochemical immunosensor to detect the H1N1 virus. This sensor is fabricated by modifying paper with a spray of hydrophobic silica nanoparticles and using stencil-printed electrodes. A glass vaporizer spray the hydrophobic silica nanoparticles (i.e., polydimethylsiloxane) onto the paper, rendering it super-hydrophobic. This essential property, super-hydrophobicity, is essential for the paper-based biosensor to operate and it is built using 30–40 spray coatings, each corresponding to a 0.39–0.41 mg/cm${}^{2}$ coating of nanoparticles on the paper. The sensor uses stencil-printed carbon electrodes modified with single-walled carbon nanotubes and chitosan to increase sensitivity. The antibodies are immobilized via glutaraldehyde cross-linking. The detection uses a differential pulse voltammetry to assess the sensitivity of the sensor at various virus concentrations that range from 10 to 104 PFU/mL. This immunosensor shows a linear behavior and selectivity for this virus with a detection limit of 113 PFU/mL. Figure 16 shows this electrochemical sensor.

#### 5.1.2. Reduced Graphene Oxide-Based H1N1 Influenza Sensor

**Figure 17:**
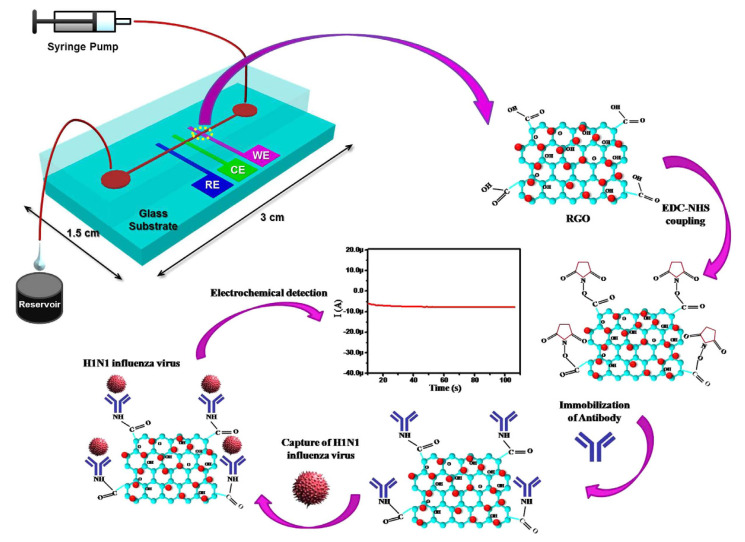
Schematic illustration of the microfluidics-integrated electrochemical immunosensing chip coated with reduced graphene oxide (RGO) [59].

Reduced graphene oxide (RGO) has recently gained a considerable attention for its use in electrochemical bio-sensing applications due to its conducting properties and large surface area. Singh et al. [59] proposed a novel microfluidic chip integrated with an RGO-based electrochemical immunosensor for label-free detection of H1N1 influenza virus. Figure 17 shows the electrochemical sensor for detection of influenza virus. Three microelectrodes are fabricated on a glass substrate using photolithography, and the working electrode is functionalized using RGO and monoclonal antibodies specific to the virus. These chips are integrated with polydimethylsiloxane microchannels. The sensor has high selectivity and a detection limit of 0.5 PFU/mL, where the chronoamperometric current increases linearly with H1N1 virus concentration, within the range of 1 to 104 PFU/mL. This microfluidic sensor provides a platform for an effective detection of biomolecules using small samples.

#### 5.1.3. Boron-Doped Diamond Surface H1N1 Influenza Sensor

**Figure 18:**
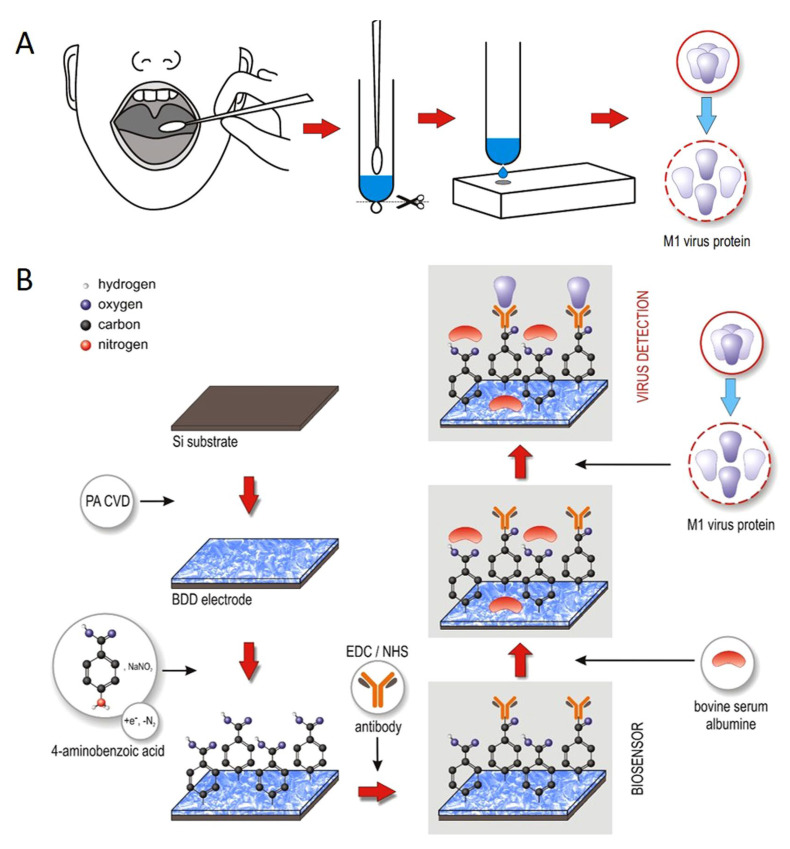
(**A**) Throat swab culture acquisition. (**B**) Boron-doped diamond electrode surface modification with polyclonal anti-M1 antibodies. The electrode identifies the universal biomarker for influenza virus, the M1 protein [60].

Nidzwoorski et al. [60] proposed a boron-doped diamond (BDD) electrode that acts as a biosensor to detect H1N1 influenza virus at ultra-low concentrations. Figure 18 shows this sensor. The BDD electrode is surface-functionalized with polyclonal anti-M1 antibodies, which serve to identify the universal biomarker of the influenza virus; M1 protein. The absorption of the M1 protein onto anti-M1 sites of the electrode changes the electrochemical impedance spectra. The sensor achieves a sensitivity of 1 fg/mL in saliva buffer for the M1 biomarker.

#### 5.1.4. Electrochemical MERS-CoV Sensor

Layqah et al. [61] proposed an immunosensor for the determination of MERS-CoV based on a competitive assay carried out on an array of carbon electrodes modified with gold nanoparticles. The recombinant spike protein S1 is used as a detection biomarker for MERS-CoV. The electrode array enables detection of different coronaviruses. The biosensor is based on indirect competition between free virus in the sample and immobilized MERS-CoV protein for a fixed concentration of antibody added to the sample. The voltammetric response is detected by a change in the peak current after the addition of different concentrations of antigen against MERS-CoV. This system uses ferrocyanide/ferricyanide as a probe to perform electrochemical measurements and square-wave voltammetry recordings. This sensor provides a linear response to concentrations from 0.001 to 100 ng/mL and 0.01 to 10,000 ng/mL for MERS-CoV and human coronavirus (HCoV), respectively. The assay takes 20 min and has a detection limit of 0.4 and 1.0 pg/mL for HCoV and MERS-CoV, respectively. The method is highly selective over non-specific proteins such as Influenza A and B.

#### 5.1.5. DNA Aptamer Immobilized Hybrid Nanomaterial-Modified Electrode H5N1 Avian Influenza Sensor

**Figure 19:**
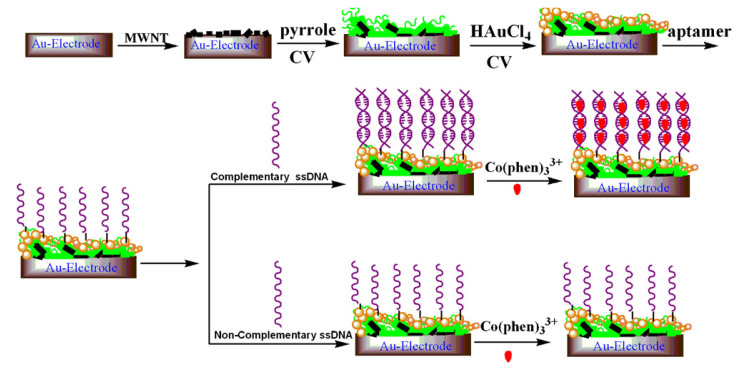
A schematic diagram showing experimental procedure of DNA aptamer immobilized hybrid nanomaterial-modified electrode [62].

Liu et al. [62] proposed a sensitive electrochemical method for the detection of avian influenza virus H5N1 gene sequence using a DNA aptamer immobilized onto a hybrid nanomaterial-modified electrode. Figure 19 shows the experimental procedure of detecting avian influenza virus H5N1 gene sequence. The modified electrode is assembled with multi-wall carbon nanotubes (MWNT), polypyrrole nanowires (PPNWs), and gold nanoparticles (GNPs) to enhance the selectivity and sensitivity. This electrode has a porous structure with a large effective surface area and high electrocatalytic activity and electronic conductivity. Due to the large surface area of the electrode, DNA aptamer is used in an amount so that the target sequence of a small quantity can be detected. The biosensor is based on the hybridization and preferred orientation of aptamer immobilized onto a modified electrode surface with its target H5N1 specific-sequence in the solution. The sensor has a detection limit of 4.3 $\times 10^{-13}$ M.

#### 5.1.6. Copper-Mediated Amplification H1N1 Avian Influenza Sensor

**Figure 20:**
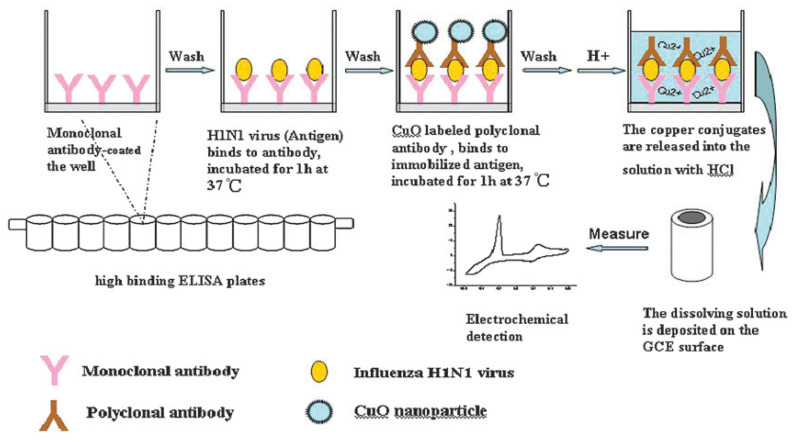
Sandwich-type immunoassay based on CuO-labeled antibody and electrochemical analysis [63].

Li et al. [63] proposed a highly sensitive electrochemical immunoassay method for the detection of H1N1 influenza virus with signal amplification by CuO nanoparticles (NPs). Figure 20 shows the electrochemical detection of avian influenza virus H1N1 gene sequence. This detection method is a copper-mediated amplified electrochemical analysis based 96-wells enzyme-linked immunosorbent assay (ELISA) plates immunosensor as a new application of sensitive, specific, and rapid detection of influenza virus H1N1. This immunoassay detects a target H1N1 influenza virus within 4 h at a concentration of 1.0 ×$10^{-12}$ g/mL, and establishes an S-curve relationship between the electrochemical signal intensity and the logarithm of the H1N1 concentration. Copper ions amplify the immune response by electrochemical detection due to their redox properties and affinity to antibodies. This immunoassay is reported useful for many applications, in particular for highly sensitive and selective detection of antigens through indirect electrochemical detection of copper ions. Its characteristics make this biosensor fit for clinical and diagnostic requirements.

### 5.2. Chemical Virus Sensor

#### 5.2.1. Gold-Carbon Nanotube Nanohybrid H3N2 Sensor

**Figure 21:**
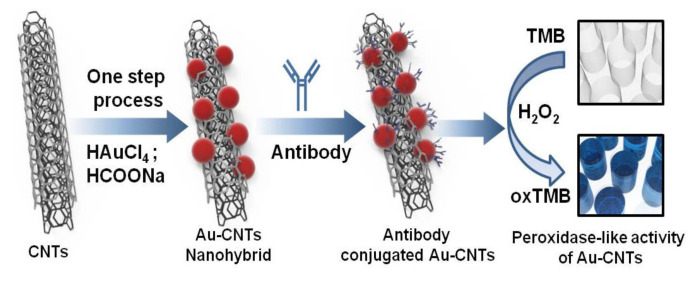
Colorimetric detection of influenza A (H3N2) virus in human serum [64].

Ahmed et al. [64] proposed to use gold (Au) Carbon nanotube (CNT) nanohybrid sensor via in-situ accumulation of Au nanoparticles (NPs) on CNTs to detect H3N2 influenza virus. They developed a colorimetric assay based on enhanced catalytic properties of nanohybrids to detect clinically isolated viruses. Figure 21 shows this chemical biosensor. The presence of this virus in the test system with specific antibody-conjugated Au CNT nanohybrids-Tetramethylbenzidine (TMB)-peroxidase H${}_{2}$O${}_{2}$ develops a deep blue color in the aqueous solution, in which the optical density depends on the virus concentration that ranges from 10 to 50,000 plague forming unit (PFU)/mL. The sensitivity of this test is also 500 times greater than that of commercial immunochromatography kits.

#### 5.2.2. Field Effect Transistor COVID-19 Biosensor

Seo et al. [65] proposed a field-effect transistor (FET) based biosensing device for detecting SARS-CoV-2 in clinical samples. The sensor is produced by coating the graphene sheets of the FET with a specific antibody against the SARS-CoV-2 spike protein. The performance of the sensor is determined using antigen protein, cultured virus, and nasopharyngeal swab specimens from COVID-19 patients. The FET device can detect the SARS-CoV-2 spike protein at a concentration of 1 fg/mL in phosphate-buffered saline. Thus, this fabrication of the sensor is a highly sensitive immunological diagnostic method for COVID-19 that requires no sample labeling.

### 5.3. Capacitive

#### CMOS Capacitive-Based Array H5N1 Influenza Sensor

Chen et al. [66] proposed a CMOS capacitive biosensor array to detect the pathogenic avian influenza H5N1 virus DNA. The sensing resolution of this sensor is significantly enhanced by a monolithic integration to reduce the parasitic effect and the use of a thin dielectric layer as the sensing interface. The array contains both planar and interdigitated electrode designs with the same CMOS readout. The interdigitated electrode design has a sensitive detection leveraged by a larger capacitance change, with a limit of detection near 10 aM level.

### 5.4. Chemiresistive

#### Chemiresistive Carbon Nanotube H5N1 Sensor

**Figure 22:**
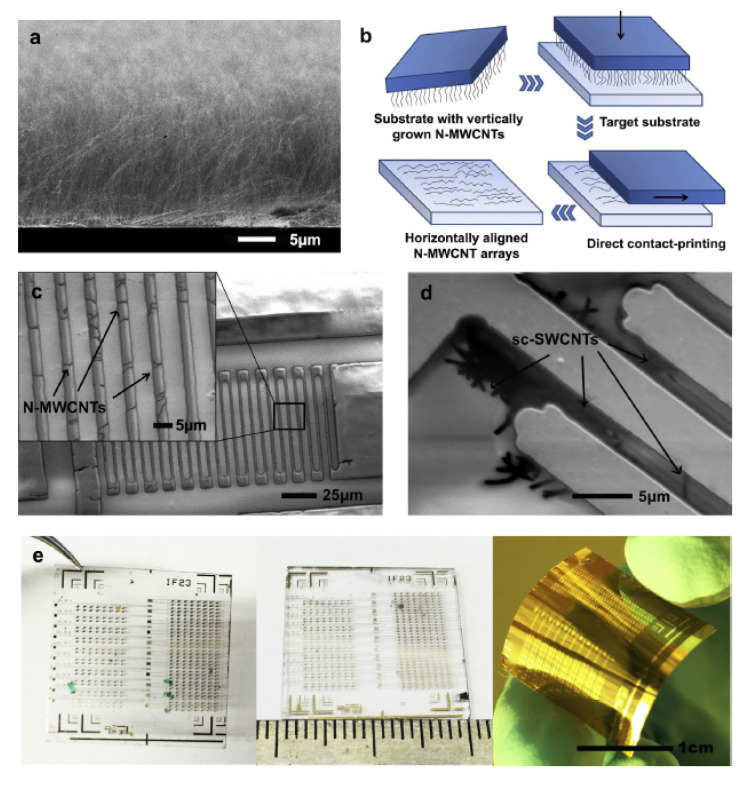
(**a**) Scanning electron micrographs of close-packed vertical arrays of N-MWCNTs grown on a Si/SiO2 substrate with CVD. (**b**) Sketch of the contact-printing process for vertically aligned CNTs. (**c**) SEM image of N-MWCNT based chemiresistor sensor device on Kapton® substrate; the higher magnified image in the inset shows a regular array of horizontally aligned N-MWCNTs bridging Cr/Au interdigitated electrodes. (**d**) Low-voltage SEM image of patterned Cr/Au interdigitated electrodes on quartz substrate bridged with sc-SWCNTs. (**e**) Optical images of CNT chemiresistor biosensor chips each containing 400 sensing devices fabricated on various substrates (from left to right: quartz, glass and Kapton® polyimide film) [67].

As shown as Figure 22, Fu et al. [67] proposed to use semiconducting single-walled carbon nanotubes (sc-SWCNTs) and nitrogen-doped multi-walled carbon nanotubes (N-MWCNTs) as two alternative active sensing elements for H5N1 virus. These sensors use long nanotubes between interdigitated metal electrodes so that individual nanotubes connect the electrodes. The nanotubes are functionalized with DNA probe sequences non-covalently attached to the sidewalls. Such functionalized-nanotube sensors can reliably detect complementary DNA target sequences of the avian influenza virus H5N1 with concentration ranging from 2 pM to 2 nM in 15 min at room temperature. The nanotube-based biosensors are small, flexible, disposable, and easy-to-fabricate and that makes them promising for point-of-care applications and clinical diagnostics.

### 5.5. Electrical

#### 5.5.1. Gold/Iron-Oxide Nanoparticle-CNT H1N1 Influenza/Norovirus Sensor

**Figure 23:**
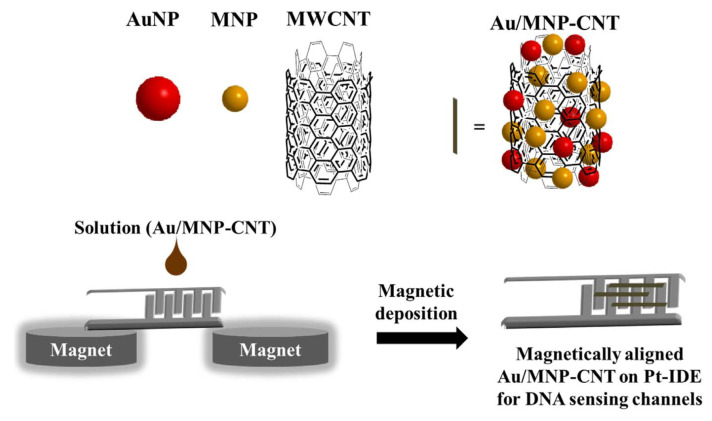
Illustration of the preparation of the magnetically aligned Au/MNP-CNTs on the Pt-IDE for DNA sensing channels [68].

Lee et al. [68] proposed a hybrid nanomaterial-based sensing platform for H1N1 influenza/norovirus detection with high sensitivity and selectivity. Figure 23 shows this electrical-based influenza and norovirus DNA sensor. The authors of this work reported that binary-NP-decorated nanotubes (bNP-CNTs) are synthesized through a two-step method and used as a bio-sensing platform. The gold/iron-oxide magnetic NP-decorated CNTs (Au/MNP-CNT) detect influenza and norovirus DNA. For this process, first, the Au/MNP-CNTs are magnetically aligned on a Pt-interdigitated electrode, and then, a thiol-group-functionalized probe DNA is attached to the Au NP surface on the bNP-CNT hybrid structure through thiol chemistry. DNA hybridization between the target influenza or norovirus DNA and probe DNA is measured to monitor an electrical conductivity change of the Au/MNP-CNTs. The sensor can detect concentrations of target DNA from 1 pM to 10 nM and has a sensitivity of influenza virus and norovirus of approximately 8.4 and 8.8 pM, respectively.

#### 5.5.2. Amperometric Bioaffinity H5N1 Avian Influenza Sensor

**Figure 24:**
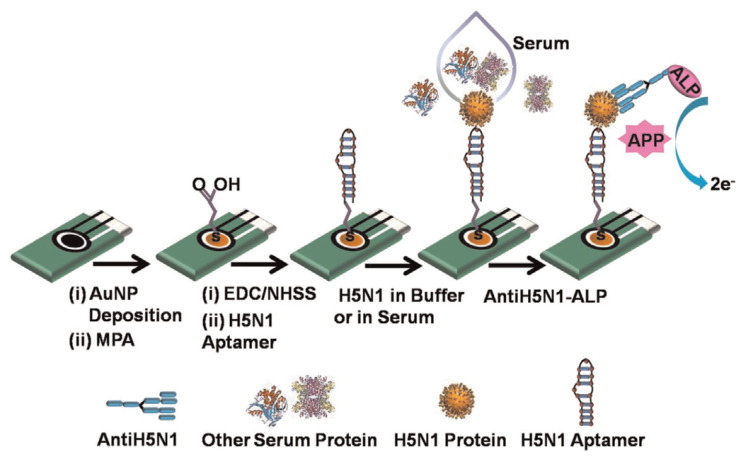
Illustration of the amperometric H5N1 detection sensor [69].

Diba et al. [69] developed a sandwich assay platform involving a surface formed aptamer–protein–antibody complex to obtain a highly selective and sensitive amperometric detection of H5N1 viral proteins using a gold nanoparticle (NP) modified electrode. This sensor is reported as the first aptamer–antibody pairing for a selective detection of H5N1. Nanoparticle deposited screen-printed carbon electrodes are functionalized by a covalent immobilization of DNA aptamer specific to H5N1 followed by adsorption of H5N1 protein. Alkaline phosphatase (ALP) conjugated monoclonal antibody is then adsorbed to form a surface-bound Au NPs-aptamer/H5N1/antiH5N1-ALP sandwich complex which reacts with the enzyme substrate 4-amino phenyl phosphate (APP). The current associated with the electrocatalytic reaction of the surface bound ALP with APP increases as the H5N1 concentration increases. The sensor detects a minimum concentration of 100 fM with a linear dynamic range from 100 fM to 10 pM using differential pulse voltammetry. Figure 24 shows the amperometric H5N1 detection sensor.

### 5.6. Optical

#### Fiber-Optic Surface Plasmon Resonance H6N1 Avian Influenza Sensor

**Figure 25:**
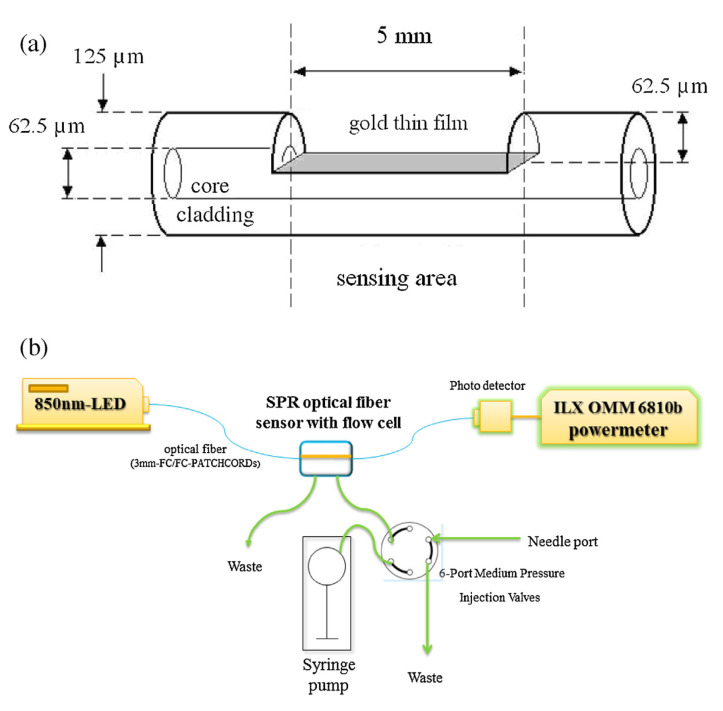
(**a**) Schematic diagram of the SPR optical fiber sensor featuring a side-polished structure. (**b**) The sensing system comprises an 850-nm LED light source, a side-polished SPR fiber in the flow cell, a six-port medium-pressure injection valve bulkhead, a syringe pump, a photo detector, and an OMM-6810B ILX power meter [70].

Zhao et al. [70] proposed a fiber optic surface plasmon resonance (SPR) sensor to detect avian influenza virus. Figure 25 shows the optical-based avian influenza sensor subtype H6. This sensor is fabricated with side-polished SPR to expose the core surface and then with a 40 nm-thin gold film deposited for the near surface sensing. The effective refractive index changes with the surface concentration or thickness of the captured avian influenza virus subtype H6. The detection surface of the SPR optical fiber sensor is prepared through the plasma modification method for chemically binding a self-assembled monolayer of isopropanol on the gold surface of the optical fiber. Subsequently, *N*-3-dimethylaminopropyl-*N*-ethylcarbodiimide/*N*-hydroxysuccinimide is activated to enable EB2-B3 monoclonal antibodies to capture the virus through a flow/injection system. The detection limit of the fabricated optical fiber sensor for the avian influenza virus is 5.14 $\times 10^{5}$ EID${}_{50}$/0.1 mL. The optical fiber sensor can be used for epidemiological surveillance and rapid diagnosis of avian influenza subtype H6.

### 5.7. Current Method for SARS-CoV-2 Detection: Real-Time RT-PCR

Currently SARS-CoV-2 RNA is detected using the qualitative Real-time Polymerase Chain Reaction (RT-PCR) test, which is intended for a qualitative detection of nucleic acid from the SARS-CoV-2 in upper and lower respiratory specimens (such as nasopharyngeal or oropharyngeal swabs, sputum, tracheal aspirates, and bronchoalveolar lavage) collected from individuals suspected of being infected with COVID-19 [71]. SARS-CoV-2 is a positive-sense single-stranded RNA virus. PCR-based methods can indicate if the virus is present or not. The assay comprises two principal steps: (1) extraction of RNA from patient specimens and (2) one-step reverse transcription and PCR amplification with SARS-CoV-2 specific primers and real-time detection with 2019 novel coronavirus (2019-nCoV) specific probes. Specimens are obtained either through nasal swab or by collecting saliva. Reverse transcription is performed using reverse transcriptase enzyme. This enzyme is used to generate complementary DNA from an RNA template. The assay targets regions of the virus nucleocapsid gene (N1 & N3) and is designed for the detection of SARS-CoV-2. The method uses PCR amplification and detection process to detect the presence of the virus. PCR amplification amplifies a segment of DNA and also Taqman chemistry, which involves a hydrolysis probe to increase the specificity of the PCR technique using fluorophore-based detection. An internal positive amplification control (IPC) is included with each specimen to ensure the absence of non-specific PCR inhibition of a sample. A sample can be interpreted as negative only if the analysis of the IPC indicates that amplification has occurred in the reaction tube but no signal from the target sequence is detected.

## 6. Discussion and Future Work

### 6.1. Discussion

These sensors were designed to detect a single component of saliva and their design is motivated for the diagnosing or monitoring of a disease. To detect the presence of saliva in the daily environment, a saliva sensor needs to detect multiple distinctive components of human saliva. These detections are performed to differentiate saliva not only from water but also from other similar fluids, such as mammals’ saliva (e.g., dog’s saliva) and human sweat.

**Saliva Component Sensors:**Table 2 summarizes the saliva component sensors. Here, electrochemical sensors are used to detect amylase, cholesterol, glucose, cortisol, and electrolyte. These sensors detect the current/potential changes between electrodes after analytes bind and interact with the working electrodes. These sensors can be easily ported and used in field tests. They may require cleaning before use. Moreover, they require liquid and sometimes large samples for detection. The component under test may need to be preserved in a liquid state, and that may be a challenge for the detection of saliva in the environment, not only because of the required sample size but also because liquid saliva may evaporate and dry out. Chemical colorimetric sensors have been used to detect urea, peroxidase, and glucose. These sensors use chemical compounds that indicate the reaction with the analytes by showing a specific color or wavelength. These sensors are portable and easy to use in the field, but are usually not reusable and need human intervention. Moreover, their accuracy range depends on the type of analyte. Therefore, these sensors cannot be used for continuous monitoring. Optical sensors have been used to detect urea and amylase. These sensors also use colorimetry or absorption of light at a specific wavelength by the analyte. They use a light source and optical probe in the test settings. They can be used for continuous monitoring. These sensors use a wavelength in the ultraviolet band and that requires safety measures to avoid harming people in the surroundings. These sensors seem to require a significant amount of hardware, but they have the advantage that no cleaning is required, and they may be used for continuous monitoring. A capacitive sensor has been used to detect mucin. This sensor measures the dielectric properties of the samples at different frequencies. While the sensor is practical, it requires maintenance; it needs to be cleaned after each use. It also requires placing the analyte in a specific location within the sensor, so it may be difficult to use it in a field test where the location of the sample may be unknown. Fluorescence-based sensors have been used to detect lysozyme. These sensors use a high energy wavelength to excite samples whose molecules absorb the light’s energy and respond by emitting light. This method is widely adopted by DNA and RNA testing in biological fields. These sensors are often used in lab environments and are usually not portable.

**Humidity Sensors:**Table 3 summarizes the surveyed humidity sensors based on their sensitivity and working principle. The surveyed sensors are based on four working principles: optical, electrochemical, chemical, and capacitive. Different from the saliva component sensors, the humidity sensors’ sensitivity is given uniformly in percentage of RH. As the table shows, the capacitive sensors are the most sensitive, followed by the optical Tungsten disulfide sensor. Other portable humidity sensors are also reported in the literature [53,54,72,73,74,75], with some of them being non-portable [76,77,78,79]. Capacitive humidity sensors are fabricated using porous or hydrophobic materials between electrodes to detect the capacitance change at different relative humidity levels. These sensors are reusable, easy to fabricate, often with a small footprint, and resistant to harsh environments. Therefore, they are good candidates for field deployments. Optical humidity sensors use optical fibers and a laser source to detect relative humidity and are often used in industrial environments. They are expensive to build as they require a specific laser source and an optical spectrum analyzer to analyze the received signal. It can provide a continuous measurement. However, these sensors are not portable. Electrochemical humidity sensors use carbon nanotubes as sensing layers between the electrodes. These sensors have a small footprint. However, their sensing range is narrower than that of the capacitive sensors. They can be reused and can be considered as candidates for field sensors.

**Virus Sensors:**Table 4 presents a summary of the reviewed virus sensors and a comparison of these sensors according to their working principle. Many of the surveyed virus sensors in this category fall within the electrochemical working principle. These sensors often adopt carbon nanotube electrodes to immobilize viruses, antibodies, or biomarker proteins and detect the electrical currents caused by changes in the concentration of the immobilized proteins of interest.

Chemical virus sensors use colorimetric assays to detect clinically isolated viruses and graphene-coated sheets on field-effect transistors, and antibody against SARS-CoV-2 spike protein to detect COVID-19. A novel sensor for SARS-CoV-2 is a chemical sensor that may also detect other coronaviruses but with the appropriate antibody. Most of these sensors use probes specific to the target virus to make it selective. Their diverse materials, sensitivity, and target virus make them difficult to compare. Moreover, they may be suitable for a laboratory setting and not for field tests. Therefore, there are plenty of opportunities to develop portable and deployable virus sensors. It is also worth noting the diverse working principles of these sensors. There are other virus sensors in the literature that are not described here [80]. Some sensors are based on chemical [81,82,83,84], electrical [85], and optical working principles [86,87]. Their operation may be similar to the ones reported here but with different properties.

### 6.2. Future Work

The surveyed sensors mostly focus on measuring a single saliva component. The presence of various components and their concentration in saliva may not only be a health indicator for detection of a disease, but also as an indicator of the risk at which people may be exposed to an infectious disease, such as COVID-19. Therefore, multi-modal sensing is required to achieve an accurate identification of human saliva. The detection of multiple saliva components in multi-modal sensing would be of great value as such sensing can offer a mechanism not only to prevent contracting COVID-19 but also other infectious diseases that are transmitted through saliva.

A drawback of existing sensors of saliva components is that they are designed for use in a laboratory environment. Therefore, it is of great interest to design portable and maintenance-free sensors that can be easily deployed in a variety of environments. The detection of human saliva is complex for various reasons: (1) many of the components of human saliva appear in small concentrations, (2) saliva that rests on surfaces may dry and dry saliva may be more difficult to sense than a liquid sample, and (3) some liquids on surfaces may be animal saliva or other human bodily fluids, such as sweat. Therefore, a sensing system may be required to detect multiple components of human saliva and those that differentiate human saliva from other possible fluids that may be found in the surroundings. For example, sweat is also liquid in its original appearance and it may also be deposited on surfaces around where people congregate. It primarily consists of water and electrolytes. The primary electrolytes contained in sweat are sodium and chloride. Sweat also contains potassium, bicarbonate, and calcium, and also amino acid and enzymes such as urea and lactate. Approximately 1% of the total volume of sweat consists of proteins, including immunoglobulins and glycoproteins. It is acidic, with a pH of 4–6. Saliva has a normal range pH of 6.2–7.6 with an average of 6.7 [88]. Resting pH of the mouth is 6.3 or higher. In the oral cavity, saliva maintains the pH at near neutrality (6.7–7.3).

As commented above, dog saliva is another possible substance that may be suspected human saliva. Dog saliva consists of mucins, carbonic anhydrase, lysozyme, testacin, lactoperoxidase, immunoglobulins, and secretory leukocyte protease inhibitor (SLPI) [89]. Sensors sensitive to these components need to be considered for building a detector of human saliva.

In lab-based saliva analysis, a proper process of sample collection requires participants to follow guidelines such as the period to avoid eating or drinking prior to sample collection. The collected samples have to be stored and preserved in proper condition before their lab analysis [90,91,92,93]. The challenge of collecting a saliva sample in an ad-hoc environment is that saliva droplets may be expelled at unknown times and fall on unknown areas. Moreover, they may dry out before they are collected, thus making it difficult for liquid-based sensors to be effectively used.

Another concern on dry saliva is that RH and hydration conditions may or may not suffice for virus survival. The observed virus survival in saliva deposited on surfaces, under a wide range of RH levels, have implications in public health, such as those reported in the case of the COVID-19 pandemic [94]. Investigation of virus survival in dried saliva is needed to determine the need of sensors that detect dry saliva.

Another challenge that sensors of saliva components face is the sensitivity required as organic components of saliva are present with very low concentrations. Moreover, saliva samples collected by sensors deployed in settings outside the laboratory may be too small for their sensitivity.

## 7. Conclusions

Because human saliva is considered a major mean of contagion of many infectious diseases, including COVID-19, this paper surveys sensors that detect or measure components of human saliva that may be used to build a saliva detection system. While the presence of saliva in a medium does not guarantee that an infectious agent is present, it may indicate a high risk of contracting an infectious disease, such as COVID-19, and it can be used to activate prevention measures.

The reviewed sensors are categorized based on their working principles. Because 98% of human saliva is water, humidity sensors may be helpful to indicate the presence of saliva. This survey presents several of these sensors and categorizes them by their working principle.

Because it may be of interest to directly identify an infectious agent in a medium, the survey also presents some of the existing sensors of viruses that spread through saliva, including influenza, MERS, and COVID-19.

This survey also discusses existing challenges and future research. Among these, the need for portable sensors that require little maintenance stands out because portability would enable a ubiquitous deployment of saliva sensors on different daily-activity settings. Some of the presented sensors detect saliva components but they are actually designed to operate in a laboratory environment. While such sensors may not be applied to the detection of saliva in the field, they may offer an insight into their operation and provide a starting point on the development of portable and practical saliva sensors.

## Figures and Tables

**Table 1:** Concentration of components in saliva [7].

Components	Concentration (μg/mL)	Percentage (%)
Amylase	476	42
Mucin	200	17
Urea	200	17
Lysozyme	9.5	1
Glucose	10	1
Cholesterol	0.14	≈0
Cortisol	0.2	≈0
Peroxidase	50	4

**Table 2:** Sensors of Organic and Inorganic Saliva Components.

Working Principle	Saliva Components	Sensor Name	Sensitivity	Sample Size
Electrochemical	Amylase	Alpha Amylase Biosensor [20]	NA	1 mL
	Cholesterol	Platinum Nano-cluster Combination Cholesterol Sensor [21]	132 μA/mMcm${}^{2}$	50 μL
	Electrolyte	Potentiometric Sensor [47]	NA	50 μL
	Cortisol	Immunosensor [22]	6 μA/(pg/mL)	NA
		Antibody Modified Gold Microarray Electrode Sensor [23]	1 pM	NA
	Glucose	Single-walled Carbon Nanotube Sensor [24]	0.24 μA.s.dL./mg	NA
		Mouthguard Cavitas Sensor [25]	0.05–1.0 mmol/L	NA
Capacitive	Mucin	CMOS Mucin Biosensors [41,42]	NA	NA
Optical	Urea	Optical Urea Sensor [39]	10.9 mg/dL	10 μL
	Amylase	Optical Amylase Sensor [40]	NA	2 mL
Chemical	Urea	Colorimetric Urea Sensor [26]	12.82%	NA
	Glucose	Colorimetric Glucose Sensor [27]	NA	NA ± 1.16 mg/dL and the mean FSG of the subjects in the diabetic group was 10.93 ± 1.93 mg/dL
	Peroxidase	ABTS Peroxidase Sensor [28]	NA	0.5 mL
Fluorescence	Lysozyme	Flourescence Lysozyme Biosensor [43]	21.8 pM	NA

**Table 3:** Comparison of Humidity Sensors.

Working Principle	Sensor Name	Sensitivity (%RH)
Optical	Tungsten Disulfide Sensor [53]	35–85
	Tin Oxide-doped Optical Sensor [54]	20–90
	Agarose-doped Polymethyl Methacrylate (PMMA) Sensor [55]	50–80
Electrochemical	Single-walled Carbon Nanotube-based Humidity Sensor [56]	20–80
Chemical	Electroceramic-Based Sensor [57]	33–95
Capacitive	Capacitive-based Humidity Sensor [50]	NA
	Alumina-based Cross-capacitive Humidity Sensor [51]	0–90
	Multi-walled Carbon Nanotube Capacitive Humidity Sensor [52]	11–97

**Table 4:** Virus Sensors.

Working Principle	Virus	Sensor Name	Detection Element	Sensitivity
Chemical	H3N2 Influenza virus	Au-CNT Nanohybrids H${}_{3}$N${}_{2}$ Sensor [64]	Monoclonal antibodies specific to H3N2 virus	3.4 PFU/mL
	COVID-19 (SARS-CoV-2)	Field Effect Transistor COVID-19 Biosensor [65]	SARS-CoV-2 antibody	1 fg/mL
Electrochemical	H1N1 Infiuenza virus	Paper-based H1N1 Infiuenza Sensor [58]	Anti Infiuenza H1N1 antibody	113 PFU/mL
		Reduced Graphene Oxide-based H1N1 Infiuenza Sensor [59]	Monoclonal antibodies specific to H1N1 virus	0.5 PFU/mL
		Boron-doped Diamond Surface H1N1 Influenza Sensor [60]	Polyclonal anti-M1 antibodies specific to H1N1	1 fg/mL
		Copper-mediated Amplification H1N1 Sensor [63]	CuO labeled polyclonal antibody specific to H1N1	1.0 $\times 10^{-12}$ g/mL
	Corona Virus (MERS)	Electrochemical MERS-CoV Sensor [61]	MERS-CoV antibody	0.4 to 1.0 pg/mL
	H5N1 Avian influenza virus	DNA Aptamer Immobilized Hybrid Nanomaterial-modified Electrode H5N1 Avian infiuenza Sensor [62]	DNA Aptamer specific to H5N1	4.3 $\times 10^{-13}$ M
Capacitive	H5N1 Infiuenza DNA	Infiuenza CMOS capacitive-based Array H5N1 Infiuenza Sensor [66]	DNA Probe specific to H5N1	10 aM level
Chemiresistive	H5N1 Influenza DNA	Chemiresistive Carbon Nanotube H5N1 Sensor [67]	DNA Probe specific to H5N1	2 pM to 2 nM
Electrical	H1N1 Infiuenza virus and Norovirus DNA	Gold/Iron-oxide Nanoparticle CNT Infiuenza/Norovirus Sensor [68]	DNA probe specific to H1N1 and Norovirus	8.4 pM to 8.8 pM
	H5N1 Avian Influenza virus	Amperometric Bioaffinity H5N1 Avian Influenza Sensor [69]	DNA aptamer specific to H5N1	100 fM
Optical	H6N1 Avian Infiuenza virus	Fiber-optic Surface Plasmon Resonance (SPR) H6N1 Avian Infiuenza Sensor [70]	EB2-B3 monoclonal antibodies specific to H6N1	5.14 $\times 10^{5}$ EID${}_{50}$/0.1 mL

## Data Availability

No new data were created or analyzed in this study. Data sharing is not applicable to this article.
